# Phase III randomized, double-blinded, placebo-controlled multicenter trial of melapuldencil-t: autologous dendritic cells loaded with irradiated autologous tumor cells (dc-tc) in gm-csf in patients with metastatic melanoma

**DOI:** 10.1186/2051-1426-2-S3-P70

**Published:** 2014-11-06

**Authors:** Robert Dillman, Andrew Cornforth, Michael Bayer, Dynesha Carbonell, Gabriel Nistor

**Affiliations:** 1 NeoStem Oncology, LLC, Irvine, CA, USA; 2 NeoStem Oncology, LLC, Encinitas, CA, USA

## Background

Genomic analyses have shown that melanoma patients have hundreds to thousands of non-synonymous mutations including unique tumor associated antigens (TAA) that can be recognized by their immune systems. The efficacy of anti-checkpoint monoclonal antibodies provides further proof that host recognition of TAA exists, but many patients do not benefit from such therapy, perhaps because they lack sufficient TAA recognition. One way to enhance TAA recognition is immunization. The best source of TAA may be autologous tumor cells that self-renew and proliferate in tissue culture (patient specific tumor stem cells). Sequential Phase II trials in metastatic melanoma patients tested the clinical benefit of repeated s.c. injections of autologous dendritic cells loaded with antigens from irradiated tumor cells derived from autologous melanoma cell lines (DC-TC), and suspended in GM-CSF. In a single arm trial 5-year survival was 50% for 54 patients treated with DC-TC. In a randomized Phase II trial, 2-year survival was 72% for 18 DC-TC patients compared to 31% in the control arm (HR = 0.27, p = 0.007). Toxicities associated with DC-TC were minimal (n = 72). Improvements in manufacturing have increased the probability of establishing a cell line and decreased the time needed to produce the product. Melapuldencel-T (DC-TC) has shown sufficient promise to receive special product assessment (SPA) and fast track designation in association with approval of this pivotal Phase III trial.

## Trial design

This is a Phase III, double-blinded, randomized, placebo-controlled trial. Eligible patients will have stage IV or recurrent stage III melanoma with at least one lesion amenable to surgical resection. Resected tumor is transferred to a manufacturing facility where a cell suspension is placed in specially formulated cell culture media, then cells from spheroids are isolated, and then expanded to at least 100 million cells. After successful establishment of a cell line and referral for treatment, 250 patients with good performance status (ECOG 0-1) will be stratified by whether they have no evidence of disease, non-measurable disease by RECIST, or measureable disease with elevated LDH or without elevated LDH. They undergo leukapheresis, and are randomized 2:1 to receive either DC-TC or autologous mononuclear cells. Both products are suspended in 500 µg of GM-CSF and injected weekly for 3 weeks, and then monthly for 5 months. The endpoint is overall survival with death from any cause the major endpoint. [NCT01875653].

